# Poor Health in Rich Countries: A Role for Open Access Journals

**DOI:** 10.1371/journal.pmed.1001543

**Published:** 2013-10-29

**Authors:** The PLOS Medicine Editors

## Abstract

In the October editorial, the *PLOS Medicine* Editors argue that the health of poor people in rich countries is of global significance and discuss why Open Access journals are particularly well suited to facilitate research and commentary on this topic.

*Please see later in the article for the Editors' Summary*

Readers may be familiar with reports of a country where economic conditions have pushed the healthcare system toward collapse. The country's government reported in 2012 that “health care spending per person has grown faster than the nation's economic output per person during the past 25 years,” and that “such rates of growth cannot continue indefinitely, because if they did, total spending on health care would eventually account for all of the country's economic output—an impossible outcome” [1]. Although the country's economy is large, its life expectancy among adults, rates of chronic disease and injuries among adolescents and children, and infant mortality all show it to be at a marked disadvantage among comparable high-income nations [2].

That country is the United States, where a recent report by the Institute of Medicine noted that “the American health–wealth paradox is a pervasive disadvantage that affects everyone, and it has not been improving” [2]. Although income is not the sole explanation for poor health indicators in the US, tens of millions of Americans, characterized in part by race and relative poverty, experience levels of health that are typical of middle-income or low-income countries [3].

The US provides a striking but by no means isolated example of how relative poverty remains a powerful determinant of health in rich countries. That other high-income countries outperform the US in health indicators and in controlling healthcare costs [4] does not change the fact that their own low-income populations experience health problems to an extent that aggregate indicators of prosperity (such as per capita income or gross domestic product) fail to predict. Among 20 Western countries, child mortality correlates strongly with the gap between the highest and lowest 20% of incomes, but not with overall health expenditures [5]. Two- to four-fold increases in mortality between highest and lowest socioeconomic strata have been reported in the UK [6]. In Greece and Ireland the implementation of austerity measures after 2007 coincided with substantial increases in suicide rates, providing tragic evidence for the health impact of economic policies that widen the gap between rich and poor [7]. To the extent that socioeconomic disparities constitute a major and growing determinant of health, we believe that general medical journals must take an active role in promoting the care of populations at greatest risk, regardless of the average income of their country of residence.

For at least three reasons, Open Access journals should lead the publication of papers on disadvantaged populations in high-income as well as lower-income countries. First, in order to attain their full impact these papers must be freely accessible to the affected public and to advocacy groups, without subscription barriers. Second, to permit adaptation and re-analysis, the results and data must be openly available without copyright restrictions.

Third, Open Access journals, by virtue of their business models, are free to prioritize papers on cost-effective, widely accessible approaches to healthcare, because these journals need not depend on the promotion of new, expensive drugs and medical devices. For closed-copyright journals, marketing strategies involving these products can generate lucrative reprint sales [8] and substantial advertising revenue [9]. However, to the extent that a journal's practices establish market demand for costly treatments of minimal incremental value, that journal contributes to the unsustainable escalation of healthcare costs. *PLOS Medicine* has, since its inception, declined to advertise pharmaceuticals and medical devices [10], leaving the journal free to make editorial decisions without competing concerns over advertising income. We believe that this policy has supported our efforts to serve as a truly global journal, in the sense that “the global in global health refers to the scope of problems, not their location. Thus… global health can focus on domestic health disparities as well as cross-border issues” [11].

Indeed, for many issues that affect the health of poor people, clear-cut distinctions between “domestic” and “cross-border” research are becoming increasingly difficult to draw, as topics of this month's *PLOS Medicine* papers illustrate [12],[13],[14],[15]. The “universal test and treat” approach—first proposed by WHO researchers based on characteristics of HIV transmission in southern Africa [16]—has informed HIV treatment recommendations and prevention programs in North America. Roadblocks to polio eradication in Afghanistan, Pakistan, and Nigeria find unfortunate parallels in Western anti-vaccination movements that have involved misinterpretations of scientific evidence in the context of political agendas. A recent editorial in *PLOS Neglected Tropical Diseases* points out that NTDs found among poor people in wealthy countries contribute substantially to health disparities, and exhibit many of the same features as NTDs seen predominantly in lower-income countries, including adverse impact on child development, pregnancy outcomes, and worker productivity [17]. At the same time, noncommunicable diseases such as diabetes and coronary artery disease, once seen as the scourges of wealthy countries, urgently require action as increasingly frequent causes of illness and death in lower- and middle-income countries [18],[19]. Whether performed in poorer or richer countries, research on diseases that disproportionately affect poor people is increasingly relevant across all countries.

According to the Institute of Medicine report cited above, “In countries with the most favorable health outcomes, resource investments and infrastructure often reflect a strong societal commitment to the health and welfare of the entire population” [2]. The *PLOS Medicine* editors agree, and we believe that Open Access journals are well positioned to strengthen such commitments by advancing practical options for improving care across the socioeconomic spectrum. As a general medical journal of global scope, we encourage the submission of papers that address clinically important problems among vulnerable, underserved, and disadvantaged populations in high- as well as lower-income countries.
